# Coronary Artery Calcium and Aging: Physiological Basis, Assessment, and Treatment Options in Percutaneous Coronary Intervention

**DOI:** 10.3390/jcdd11070224

**Published:** 2024-07-15

**Authors:** Mohamed Abdirashid, Umberto Barbero, Chiara Cavallino, Ludovica Maltese, Elodi Bacci, Danilo Reale, Giorgio Marengo, Michele De Benedictis, Francesco Rametta, Fabrizio Ugo

**Affiliations:** 1 Sant’Andrea Hospital, 13100 Vercelli, Italy; 2 Santissima Annunziata Hospital, 12038 Savigliano, Italy; umberto.barbero@unito.it (U.B.);

**Keywords:** percutaneous coronary intervention, calcium, coronary artery disease

## Abstract

Coronary artery calcification is a complex anatomical and histological pathology with different pathways that contribute to calcium deposit and calcification progression. As part of the atherosclerotic process, extensive calcifications are becoming more common and are associated with poorer PCI outcomes if not properly addressed. Since no drug has shown to be effective in changing this process once it is started, proper knowledge of the underlying pathogenesis and how to diagnose and manage it is essential in contemporary coronary intervention. Atherosclerosis is a pandemic disease, quickly spreading across the world and not limited anymore to the industrialized Western world. In this paper, we review the role of intracoronary imaging and the main technologies available and propose a simple and rational algorithm for the choice of a preferential first strategy in the treatment of severely calcified coronary atherosclerosis, followed by three emblematic cases on how we successively applied it.

## 1. Introduction

Coronary artery calcification (CAC) is the deposition of calcium in the walls of coronary arteries as part of the process of plaque formation and atherosclerosis [1]. The process of coronary artery calcification (CAC) formation entails the buildup of lipids, immune cells, and other substances within the arterial wall. This accumulation initiates an inflammatory response, ultimately resulting in the deposition of calcium. The prevalence of CAC varies with age and gender, affecting nearly 90% of men and over 65% of women aged over 70 years [2]. And, as part of the spectrum of atherosclerosis, the clinical consequences can range from asymptomatic to chest pain (angina) and shortness of breath up to life threatening ones such as heart attacks and myocardial infarction [3].

Diagnosing coronary artery calcification (CAC) is crucial in contemporary percutaneous coronary intervention (PCI), and it can be achieved using both invasive (i.e., intravascular) and non-invasive imaging techniques. These imaging methods enable the visualization of coronary arteries and the identification of CAC, facilitating the planning and optimization of PCI procedures. Various tools are indeed accessible for managing CAC in modern PCI, such as cutting balloons, atherectomy techniques, and coronary lithotripsy [4,5,6,7].

In modern PCI, the selection of a tool for managing coronary artery calcification (CAC) hinges on factors like the severity and location of the calcification as well as the patient’s unique anatomy and other considerations. The purpose of this review is to consolidate the latest evidence regarding CAC pathophysiology, diagnosis, and treatment.

## 2. Histopathological Finding in Aging Coronary Artery Disease

Aging represents a significant risk factor for the onset of coronary artery disease (CAD), marked by the gradual narrowing and stiffening of coronary arteries due to the deposition of lipids, inflammation, and calcification [1]. Inflammation stands as a pivotal characteristic of CAD and is believed to exert a substantial influence on disease progression [8]. Inflammatory cells like macrophages and T cells reside within the arterial wall, secreting cytokines that foster the recruitment of additional immune cells, thereby contributing to the formation of advanced lesions. Histopathological observations in aging CAD typically commence with fatty streaks, progressing to fibrous plaques, complicated plaques, and eventually calcifications. The initial lesion in CAD manifests as a fatty streak, characterized by the accumulation of lipids—chiefly cholesterol and triglycerides—within the intima layer of the artery wall [9]. As fatty streaks progress, fibrous plaques emerge, characterized by the accumulation of smooth muscle cells, connective tissue, and extracellular matrix. Typically stable, fibrous plaques often do not induce significant symptoms. Conversely, complicated plaques denote more advanced lesions, marked by the accumulation of necrotic debris, inflammation, and calcification [8,9]. Calcification, a prevalent feature of aging CAD, entails the deposition of calcium within the arterial wall. This process can rigidify and diminish the flexibility of arteries, potentially heightening the risk of plaque rupture and thrombosis. Consequently, thrombus formation—a blood clot—can ensue, leading to severe cardiovascular events such as a heart attack.

### Molecular Mechanism of Coronary Artery Calcification and Its Clinical Interplay

The molecular mechanism of CAC is complex and not fully understood. However, it is thought to involve a combination of factors including inflammation, oxidative stress, and altered mineral metabolism [1,10].

Inflammation serves as a pivotal factor in both the initiation and progression of coronary artery calcification (CAC). Immune cells, notably macrophages, are recruited to sites of arterial injury or inflammation, where they undergo activation and release cytokines along with other signaling molecules. Cytokines like Tumor Necrosis Factor-α (TNF-α) and Interleukin-1 β (IL-1 β) further amplify immune cell recruitment and contribute to the formation of atherosclerotic plaques [11].

Moreover, oxidative stress contributes to CAC by fostering the generation of reactive oxygen species (ROS), which inflict damage upon cells and tissues. ROS activation can stimulate pro-inflammatory signaling pathways and trigger the expression of genes involved in mineral metabolism and bone formation [12]. Additionally, perturbations in mineral metabolism may exacerbate CAC by fostering the deposition of calcium and phosphate within the arterial wall.

Some clinical characteristics increase the risk of developing CAD, such as chronic kidney disease and diabetes mellitus (which frequently overlap with aging). Chronic kidney disease (CKD) fosters vascular calcification in many ways: elevated phosphate levels in the blood lead to vascular deposits and stimulate osteogenic transitions in vascular smooth muscle cells (VSMCs). As CKD progresses, kidney functions decline, resulting in further imbalances of phosphorus, calcium, and magnesium, which are closely linked to calcification. Hypercalcemia and hyperphosphatemia in hemodialysis patients further accelerate CAC, highlighting the need to maintain normal serum calcium and phosphorus levels. In advanced CKD, high parathyroid hormone (PTH) secretion affects pro-inflammatory cytokines and vascular remodeling: elevated PTH levels transform VSMCs into osteoblast-like cells, leading to atherosclerosis and vascular calcification. High PTH secretion also activates protein kinase pathways and enhances advanced glycation end products (AGEs) and Interleukin-6 expression. Additionally, uremic toxins accumulate in CKD patients due to a decreased estimated glomerular filtration rate (eGFR), causing clinical symptoms. These toxins trigger immune disorders and inflammatory damage, directly increasing vascular calcification risk in end-stage renal disease patients [12,13,14,15]. The role of vitamin D deficiency is instead controversial: despite the fact that its scarcity may enhance pro-inflammatory factors, mediating calcification, up-to-date studies show contrasting results, necessitating further investigation into the vitamin D and vascular calcification relationship [16].

If with aging the percentage of CAC relative to plaque burden increases progressively in both individuals with type 2 diabetes and those without diabetes, in subjects with either type 1 or type 2 diabetes, coronary lesions show an increase in areas of severe CAC [14]. This association between diabetes and vascular calcification is driven by an active pro-inflammatory and pro-osteogenic program: diabetic patients face a higher risk of endothelial cell dysfunction (ECD), and diabetes-related hormonal and physiological abnormalities, such as oxidative stress, endothelial dysfunction, changes in mineral metabolism, increased production of inflammatory cytokines, and the release of osteoprogenitor cells into the bloodstream, can promote intimal calcification. Hyperglycemia further exacerbates oxidative stress by increasing glucose oxidation in the citric acid cycle and activates the protein kinase C (PKC) pathway by enhancing diacylglycerol synthesis, which in turn activates protein kinases C-β, -δ, and -α [15].

Finally, as the arterial wall undergoes remodeling in response to injury or inflammation, there is a breakdown of the extracellular matrix and the release of calcium-binding proteins such as osteopontin and matrix *Gla* protein [17]. These proteins can bind calcium ions and facilitate their deposition in the arterial wall.

## 3. Diagnostic Tools to Detect Coronary Artery Calcifications

Several diagnostic tools are available for detecting coronary artery calcification (CAC) starting from a simple baseline angiography, but the calcium burden can be better defined by coronary computed tomography (CCT), intravascular ultrasound (IVUS), and optical coherence tomography (OCT).

**Figure 1:**
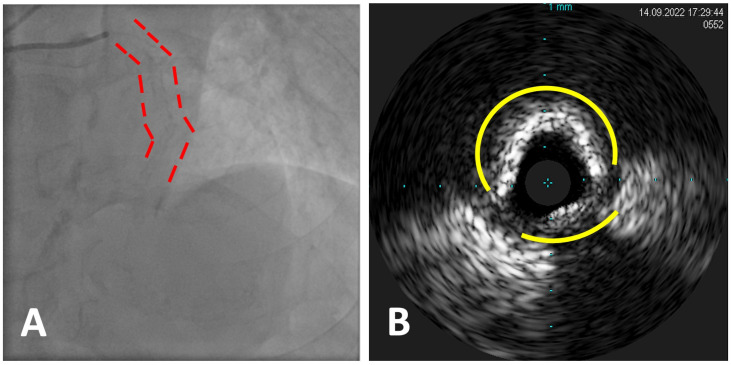
Angiography appearance (**A**) and IVUS imaging (**B**) of a calcified plaque with a >270° calcium arch with a single bright wall without any evidence of what is behind.

During invasive coronary angiography, it is possible to detect the presence of calcified plaques, and angiographic CAC is often classified into three groups: none/mild, moderate, and severe. While mild calcifications could be hard to visualize, moderate calcifications are defined as radiopacities noted only during the cardiac cycle before contrast injection, and severe calcifications are suspected when radiopacities are seen without cardiac motion before contrast injection, usually affecting both sides of the arterial lumen in a tram-line fashion [18] (Figure 1A).

Computed tomography (CT) stands as a non-invasive diagnostic tool capable of quantifying coronary artery calcium (CAC) during an ECG-gated CT scan, expressing its presence through a CAC score derived from the amount and density of calcified plaques within the arteries. CT scanners exhibit high sensitivity and specificity in detecting calcium, with the sensitivity diminishing and specificity increasing as the CAC score rises, enhancing the predictive capacity for coronary artery disease (CAD) [19,20]. When the CAC score surpasses 0 Hounsfield units (HUs), it suggests the presence of atheroma, while scores exceeding 400 HUs indicate a need for further diagnostic assessment, typically involving invasive coronary angiography. Notably, the CAC score holds prognostic value in predicting cardiac death and myocardial infarction (MI). Prospective registries have revealed that asymptomatic individuals with CAC scores exceeding 400 HUs exhibit a poorer cardiovascular prognosis, even in the absence of traditional risk factors, compared to those with over two risk factors but without CT-detected calcium [21], making a coronary CT scan with CAC score evaluation a reasonable tool for cardiovascular risk assessment in asymptomatic patients at intermediate risk.

Among invasive tools, intravascular assessment of calcium is possible using intravascular ultrasound (IVUS) and optical coherence tomography (OCT).

IVUS can help detect coronary calcium by identifying the presence and extent of calcified plaque bright echoes with acoustic shadowing within the coronary artery walls with a sensitivity of 90% to 100% and a specificity of 99% to 100% [22]. This technique can also provide information on plaque morphology, which can help guide the selection of optimal treatment strategies. According to IVUS evaluation, the coronary calcium deposit may be defined as superficial if present in the intima to lumina boundary or deep when it is found within the medial–adventitial border or even closer to the adventitia. Similarly, calcium burden can be defined both in terms of degrees of vascular involvement (with significant degrees being more than 180°, severe being more than 270°, and circumferential when encompassing the whole 360° arch of the vessel) and in terms of length of the calcified plaques [23]. Despite the limitation of ultrasound, which does not penetrate calcium, restraining a precise evaluation of calcium volume, calcium thickness can be inferred by the presence of reverberating artefacts behind the calcium wall (which usually identifies thinner deposits) [24] or on the opposite by the evidence of a single bright wall without any evidence of what is behind (highly suspicious for thick calcium) [25] (Figure 1B).

Optical coherence tomography (OCT) is an intravascular imaging technique that uses light waves instead of ultrasounds to produce higher-resolution images of the internal structures of coronary arteries (10 to 20 µm compared to 150 to 200 µm of the IVUS) with similar sensitivity (95% to 100%) and specificity (97% to 100%) [26,27]. The main advantage of using OCT to investigate calcified arteries is the highly informative evaluation of calcium morphology, including its thickness, surface characteristics, optical density, and depth within the arterial wall: indeed, because light penetrates calcium, OCT can also assess calcium thickness and define calcium volume.

## 4. Treatment Options for Coronary Artery Calcifications

No medical therapy has yet shown to stop CAC [28], and furthermore, it is well known that CAC increases the chances of procedural failure and complications during balloon angioplasty [29]. It is therefore mandatory to address calcium with a well-defined strategy and treatment algorithm in mind. The simple approach with a standard non-compliant balloon indeed often requires high-pressure dilations with unequal force applications due to different and unpredictable amounts of calcification across the lesion length, carrying the risk for dissection and acute vessel closure, slow flow, MI, and perforations [30]. Even when these problems are overcome, a high calcium burden may impede proper device delivery and lead to stent under-expansion and malapposition, which in turn pave the way for restenosis and stent thrombosis [31].

The outcomes of calcified plaques stented with DES seem to be as good as in non-calcified plaques, provided the lesion is adequately prepared and the stent is correctly deployed [5], since potential risk factors for restenosis and repeat revascularization on top of stent under-expansion and malapposition include damage of DES polymer coats by calcified lesions or the aggressive use of debulking devices (such as rotational atherectomy) that might directly promote neointimal hyperplasia [32].

In the last year, the interventional community has moved between a debulking approach and a plaque-modification philosophy. This shift has been driven by the increasing experience in coronary calcium treatment but also thanks to the growth of calcium-dedicated tools. Several device options are available today for managing coronary artery calcification during percutaneous coronary intervention (PCI). These devices include dedicated high-pressure non-compliant balloons, cutting balloons, scoring balloons, atherectomy devices (such as rotational atherectomy, orbital atherectomy, and excimer laser atherectomy ones), and intravascular lithotripsy.

High-pressure balloon inflation devices use a high-pressure inflation technique to modify calcified plaques without causing significant arterial wall traumas other than barotrauma [33].

Cutting balloons have micro-blades on the surface that can cut through calcified plaques and modify the lesion before stent implantation [34].

Scoring balloons have a similar aim, which is pursued using small notches on the surface of the balloon that create a series of micro-cuts in the calcified plaque.

The aim of these balloons is not to remove calcium or to debulk the plaque, but they are mostly used to create a “controlled dissection” leading to better plaque preparation in order to achieve a greater expansion with the subsequent non-compliant balloon dilations. They are used more on fibrotic and moderately calcified plaques and have limited use in highly calcified lesions.

The concept of plaque ablation instead traditionally involved the disruption of the calcified superficial portion of the plaque into microscopic, subcellular-sized fragments by means of rotating burrs or a photoacoustic mechanism (i.e., laser). Rotational atherectomy (RA; Boston Scientific, Boston, MA, USA) tools use a high-speed, diamond-encrusted elliptical burr to ablate calcified plaques through rotation: the burr can reach up to 200,000 rpm, scraping the hard calcified cap of the plaque while deflecting off of softer elastic tissue (the principle of differential cutting) [35]. The small, nanometric particles of calcium (<10 µm) ablated from the stenosis enter the blood flow and are eliminated by the reticuloendothelial system, but it is important to perform a short round of ablation with adequate (30 s at least) stops to let the normal flow restore and avoid excessive heating of the burr. Indeed, good practices size the burr–artery ratio as 1:2, using a pecking motion during the ablation session, restricting ablations to <20 s and retracting the burr if decelerations >5000 rpm during ablation occur [36].

Interestingly, in the pre-stent era, the use of RA alone was associated with intensified neointimal hyperplasia post-PCI, probably due to platelet activation and thermal injury, stressing the need to cover the ablated plaque with antiproliferative drugs by means of a drug-coated balloon or drug-eluting stents. RA carries an increased risk of periprocedural complications (dissection, slow flow, MI, perforation, and burr entrapment), which must be known and prevented by means of the abovementioned attentions.

Because of inherent risks and costs related to RA, without clear evidence of benefits compared with a proper balloon preparation, RA is a reasonable strategy in calcified lesions that are not crossable by a balloon catheter or by IVUS or that are not adequately dilated before stent implantation (i.e., the classic dog-bone appearance). Furthermore, the use of RA has moved itself from a pure debulking strategy to a gentler plaque modification philosophy. Contraindications to RA use are the presence of thrombus, the stenosis being located in a saphenous vein graft, or prior vessel dissection [36]. However, although it is off label, there are data of RA use in STEMI patients as a bail-out strategy with low in-hospital mortality [37].

Orbital atherectomy (OA; Diamondback 360, Cardiovascular Systems, St. Paul, MI, USA) devices use a high-speed rotating diamond-coated crown (instead of a burr) mounted on the coil, which consists of three helically wound wires that can be compressed with the application of pressure like a spring to ablate calcified plaque in a circular motion. The OA crown orbits over the atherectomy guidewire in an elliptical path, exerting a centrifugal force on the vessel wall as well as a differential ablative effect on hard and soft surfaces, producing particles <2 µm in size. This technology allows both superficial sanding and cracking of deep calcium effectively working on both superficial and deep components of the calcified plaque. The rotational speed can be controlled to progressively generating a larger orbit of rotation, thus becoming substantially larger than the cross-sectional area of the rotating element adapting the single available crown to different vessel diameters in a one-fits-all fashion [38].

In contrast with the previous mentioned calcium debulking techniques, the aim of intravascular lithotripsy (IVL; Shockwave Medical, Fremont, CA, USA) is to fragment the entire calcium accumulation of the vessel, moving from a single long circumferential totally calcified plaque to a set of fragments capable of moving and adapting to the subsequent balloon dilatation of the lumen [4,39]. This device is composed of a monorail balloon dilatable catheter that has a dual port hub: one is attached to the balloon indeflator and the other one to the pulse generator through a connector cable. Once activated, it delivers acoustic pulse waves circumferentially to disrupt superficial and deep calcification. This increases the compliance of the calcific plaque just as when a single large boulder is reduced to gravel. Compared to RA and OA, IVL reduces the risk of complications such as balloon slippage and arterial wall injury [40].

Finally, excimer laser coronary atherectomy devices (ELCA; Spectranetics, Colorado Springs, CO, USA) use focused laser energy to generates transient high-pressure waves that vaporize calcified plaques through a photoacoustic mechanism [41]. ELCA uses xenon-chloride gas as a medium to generate ultraviolet pulses at a standard 308 nm laser range. These pulses exert their effect through three different mechanisms: a photochemical effect (the pulses destroy carbon bonds of the atherosclerotic and vascular material); a photothermal effect, which means that intracellular water is heated until it causes cellular destruction; and a photomechanical effect, mediated by the heat bubbles formed at the tip of the catheter exploding and damaging the vascular plaque [31]. To date, the most common indication for its use is a balloon or microcatheter uncrossable lesions, but this technique may be used in a wide spectrum of cases such as with organized thrombus, in-stent restenosis, and acute or chronic stent under-expansion.

Excimer laser atherectomy is normally performed with a continuous saline infusion, but its use with blood and contrast has been reported to be successful, especially for stent under-expansion. The use of blood or contrast creates larger imploding bubbles with a consequent amplified acoustic–mechanical effect that increases the plaque debulking effect along with the risk of major intraprocedural complications [42].

Despite its unique ability to overcome balloon or microcatheter uncrossable lesions, its use in clinical practice is still confined due non-negligible intraprocedural complications such as vessel dissection (especially with superficial calcium) and perforation [43] and due to the potential risk of wire disruption, especially when used over a polymer-jacketed coronary wire [44].

## 5. Need for an Algorithm

Despite the plethora of technologies available, calcified lesions remain one of the most complex and difficult type of lesions to treat, leading frequently to poor outcomes. For this reason, many algorithms for the treatment of calcified lesions have been proposed, many using imaging as a starting point, or the inefficacy of previous treatments to navigate through the options. However, the use of imaging is low in real-world practice. It is a little bit higher when we are using “new” technologies like OR, where in recent registries the prevalence of imaging was only 55%. Moreover, the use of imaging drops to negligible levels when the debulking technique is old and the operators are more confident in using it, with a prevalence of 6.9% in large European registries. This is not only due to the fact that it is not possible to use imaging devices in uncrossable lesions, since imaging has not been used even after the stenting [45].

Moreover, we believe that the choice of a preferential first strategy is pivotal in simplifying this complex procedure and reducing the rate of complications.

**Figure 2:**
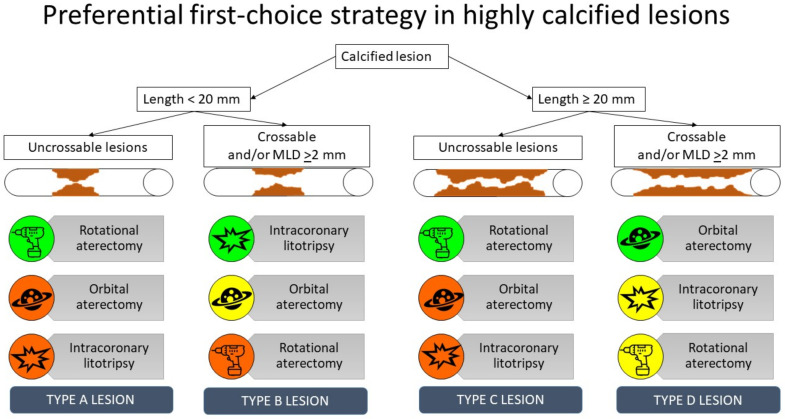
The algorithm for the choice of the preferential first-choice strategy in the treatment of highly calcified lesions.

For this reason, we propose an operative algorithm that comes from real-world experience with the technologies available (Figure 2).

### 5.1. The Algorithm

When facing a calcified lesion, many factors need to be accounted for, starting from the choice of the guiding catheter. As in all complex PCIs, we need to secure an adequate setting with enough support and sufficient caliber to accommodate the materials and to deliver them through calcified lesions.

After the choice of the guiding catheter and the wire crossing of the lesion, we are guided in the preferential first choice strategy by two factors:

1. The length of the lesion (and in long lesions, the tortuosity).
2. The MLD of the calcified lesion, especially if it is crossable or uncrossable.

The definition of uncrossable lesions is a controversial topic, since for some it is a lesion not crossable by the imaging probe, but for others it is uncrossable by balloons or by a microcatheter. In our practice, we define all the above scenarios as uncrossable lesions.

The available technologies for the treatment of severely calcific coronary atherosclerosis are as follows:

- Rotational atherectomy (RA);
- Orbital atherectomy (OA);
- Intravascular lithotripsy (IVL).

Excimer laser coronary atherectomy (ELCA) is not widely available, and the Food and Drug Administration (FDA) currently approved indications for excimer laser coronary angioplasty do not include highly calcified lesions.

Cutting/scoring balloons should be used only in moderately calcified lesions, and the available data suggest they are ineffective in highly calcified lesions [46].

The use of OPN in highly calcified lesions is controversial, and it is mainly used as a bail-out or second-choice strategy [47,48].

We gave the three main techniques a color rating according to how useful they are in the different scenarios:

- Green: preferential first choice;
- Yellow: second choice or useful in a specific subset;
- Red: futile or potentially harmful.

As in the algorithm (Figure 2) in Type A lesions (short lesions < 20 mm with a tight MLD, especially if uncrossable), our first choice is RA. In this type of lesion, there is frequently a difficulty in crossing the lesion with the materials, and for this reason, we do not suggest using IVL as a first choice. Moreover, we do not recommend OA in very tight uncrossable lesions since the 5–6.5 mm nose of the device may not cross the lesion, and consequently the crown would not be able to debulk the calcium. Furthermore, if pushed in an uncrossable lesion, it may become dangerous, as we describe in the first case below. In fact, if we push the Diamondback against an uncrossable lesion, the ellipses of the device will increase, and it may cause a dissection of the vessel before the uncrossable lesion. Conversely, RA has the peak of its debulking effect on the tip of the device, remaining effective even in very tight uncrossable lesions and in CTOs.

In Type B lesions (<20 mm and crossable lesions, with an MLD ≥ 2), our first choice is IVL, especially when the calcium distribution is concentric, since it allows an effective cracking of the calcium. OA is effective in this type of lesion, and it can be an alternative to IVL in eccentric lesions, although in our opinion IVL is the best first choice since it will allow an easier and effective preparation of the lesion in most Type B lesions. We do not recommend RA in this setting, especially if the MLD ≥ 2 mm for similar reasons and since it would be necessary to use a large burr to address this lesion and it would be ineffective in most cases.

In Type C lesions (>20 mm long and tight with an MLD < 2, especially if uncrossable), our first choice is RA, especially in very tight lesions, although in long lesions there is a higher risk of bur entrapment. Counterintuitively, using undersized burrs can increase the rate of this event. In the case that the lesion is not so tight and it allows the passage of the nose of the OA device, this can be a very good first choice since it does not require the change in the caliber of the bur. For this reason, OA is our first choice in Type D lesions (>20 mm long and not so tight, crossable lesions, especially if with an MLD ≥ 2 mm). Moreover, in long lesions we do not use IVL as first choice since, as opposed to focal lesions, there is a higher probability not to be able to treat the lesion in its entirety with one balloon.

After the use of any of the previous techniques, we suggest further preparation of the lesions with NC balloons and the use of imaging (IVUS or OCT) to confirm the quality of the lesion preparation.

As a point of interest, there are scarce data about the cost effectiveness of these devices; nonetheless, the importance of this topic increases everyday due to the worldwide crisis affecting many healthcare systems. In a couple of studies on orbital atherectomy, despite the higher initial cost of the device, it performed well, even when compared to orbital atherectomy [49,50]. In a paper by Garrison et al., when compared to the standard treatment, orbital atherectomy is likely to be cost effective and was projected to be cost saving in an inpatient setting [51]. No data are available for intravascular lithotripsy [52].

### 5.2. Case 1

**Figure 3:**
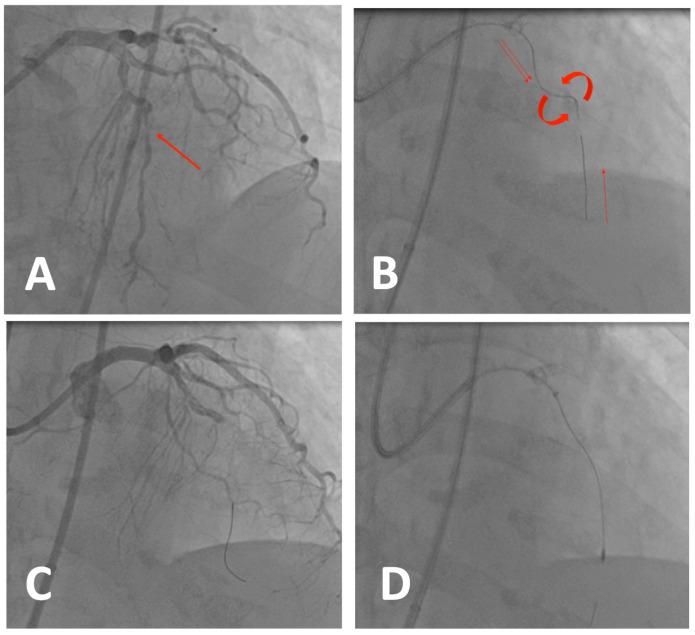
Case 1. Left coronary artery with left anterior descending (LAD) artery subocclusive stenosis (**A**). Orbital atherectomy attempt on LAD: The nose of the device is not crossing the lesion and the crown is not working. We can observe the tension from pushing the device and the ViperWire getting retracted (**B**). Dissection of proximal LAD and complete occlusion of the vessel. The patient had ST elevation and is unstable (**C**). Switch to RotaWire with microcatheter and rotational atherectomy (**D**).

An 83 y.o. woman with hypertension and obesity was referred to our center for unstable angina and a slight reduction in the ejection fraction. The coronary angiogram (CAG) showed severe three-vessel coronary disease:

- Multiple subocclusive calcified stenoses of proximal and mid LAD and a CTO of the first diagonal (Figure 3A).
- Multiple subocclusive calcified stenoses of all the Cx with an 80% ostial stenosis of a big OM.
- Subocclusive calcified stenosis of the distal RCA and proximal PDA.

After discussing the case, we decided to perform a two-step revascularization of Cx and the LAD. We started with the Cx: we positioned a BMW guide in the OM and a Sion black in the distal Cx. Since the Cx was slim and with a small distribution, we decided to treat the CX/OM axis with a provisional strategy. However, it was impossible to cross the lesions with the balloons, so we used a child-in-mother Guidion 6F and a microcatheter Corsair pro XS to exchange the BMW with a RotaWire and performed an RA with 1.25 and 1.5 burrs on the Cx/OM axis. Afterward, it was relatively easy to prepare the lesions with a 2.5 mm NC balloon, implant a 2.5 × 38 mm DES, and post-dilate it with 3 and 4 mm NC balloons. We did not recross the distal Cx since it had a TIMI III flow.

After a week, we performed the second procedure on the LAD. The planned strategy was a revascularization with OA. Therefore, we engaged the LM with a 7F EBU 3.75 guiding catheter and placed the ViperWire in the distal LAD. Afterwards, we tried to treat the lesion, but it was impossible to cross the second stenosis with the nose of the device (Figure 3B). As explained above, the crown of the device is placed 6.5 mm behind the nose, and by pushing the device on the uncrossable subocclusion, there is a high risk of dissecting the vessel. In this case, the patient had an ST elevation with chest pain, and the angiogram showed a dissection of the proximal LAD (Figure 3C). This is a challenging scenario: a flow-limiting dissection before a subocclusive and uncrossable calcified lesion. Since there was enough space between the stenosis and the dissection, we gently expanded a balloon on the dissection to try to give flow. After that, we decided to switch to an RA strategy. Therefore, we tried to place a microcatheter distally to exchange the Viperwire with a RotaWire Floppy. After that, we tried to pass the lesion with a 1.25 mm burr (at 170 rpm) with no success due to the insufficient support of the guiding catheter. For this reason, we placed a guideliner 7F close to the lesion and successfully crossed the lesion with the 1.25 mm burr (180 rpm, 2 min of active atherectomy in total) (Figure 3D). We then performed an IVUS-guided stenting of the LM and proximal and mid LAD with three DESs (2.5 × 24 mm, 3 × 28 mm, and 5 × 12 mm) and a DCB on the distal LAD, followed by IVUS-guided post-dilatation. There was a guide-induced perforation of the distal LAD that we treated with two micro-coils. The patient was stable and with stable and asymptomatic mild pericardial effusion and was discharged at home after observation in the ICU.

### 5.3. Case 2

**Figure 4:**
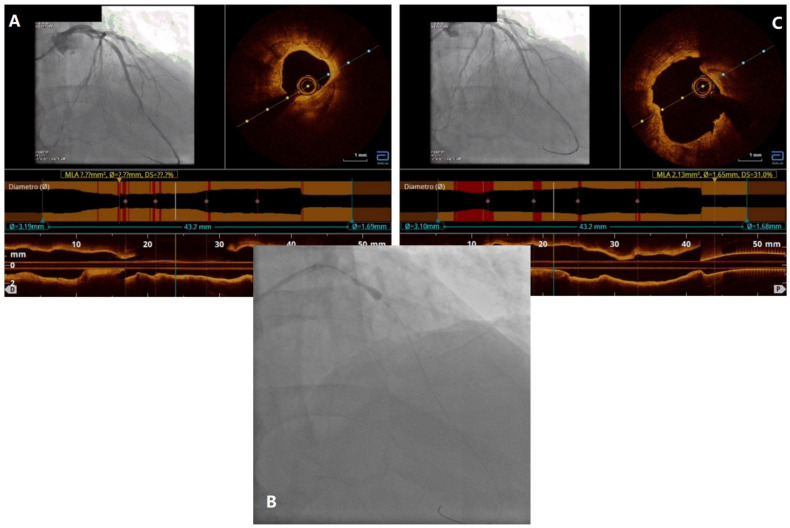
Case 2. OCT scan showing a 270° thick calcium arch (**A**); subsequent intravascular lithotripsy (IVL) with a 4.0 × 12 mm shockwave balloon (**B**); OCT scan after IVL and predilatation: we can see the cracking of the calcium and an adequate lumen (**C**).

A 72 y.o. former smoker man with dyslipidemia and diabetes was referred to our center with NSTEMI. The coronary angiogram showed a CTO of a small RCA and a subocclusive and thrombotic stenosis of the mid LAD. After successive thrombus aspiration, we performed an OCT scan that showed a long and highly calcified stenosis with a 270° arch (Figure 4A). Following our algorithm, we decided to perform an IVL with a 4 × 12 mm shockwave balloon (Figure 4B). The second OCT scan showed successful cracking of the calcium (Figure 4C) that allowed the implantation of a 4 × 28 mm DES following post-dilatation. The final OCT scan confirmed the good result of the stenting. The patient was discharged at home 4 days later with no complications.

### 5.4. Case 3

**Figure 5:**
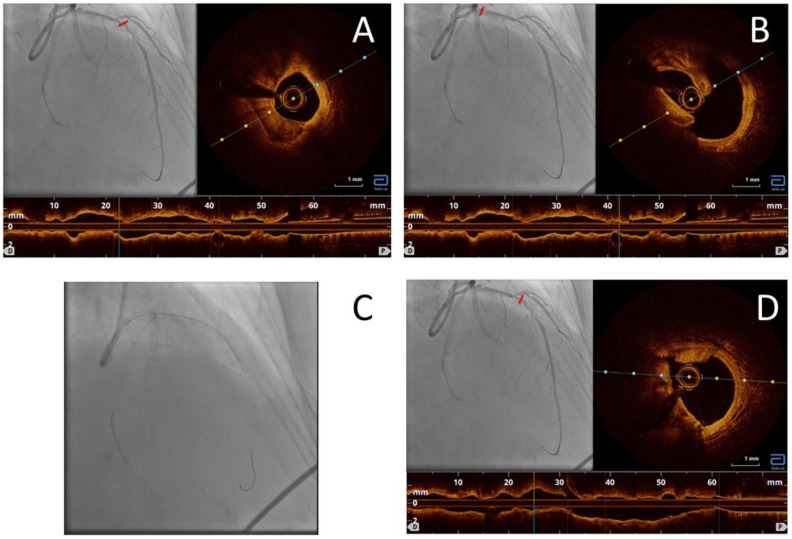
Case 3. OCT scan showed calcific plaque of mid left anterior descending (LAD) artery (**A**); OCT scan showed an emptied plaque of proximal LAD (**B**); orbital atherectomy on LAD (**C**); good result of atherectomy on LAD (**D**).

A 67 y.o. former smoker woman with dyslipidemia, diabetes, and mild CKD was referred to our center for unstable angina with anterior T-wave inversion. The echocardiography showed antero-apical hypokinesia with 50% EF. Therefore, we performed a coronary angiogram that showed a diminutive RCA; a virtual LM with an almost-separated origin of the LAD and Cx; a CTO of OM in a dominant Cx; and a long and calcified critical stenosis of the proximal and mid LAD with a proximal stenosis that looked like an ulcerated plaque. Since we had a highly calcified critical stenosis and a CTO, requiring a complex PCI, we decided to schedule the procedure in a second day. We started the second procedure with the CTO: after crossing the occlusion with a Sion black by a FineCross microcatheter, positioning a BMW in the distal Cx, and multiple predilatations of the stenosis, we implanted two DESs on the CX-OM axis. We left the jailed BMW in the Cx and we tried to wire the LAD with a Sion black wire unsuccessfully due to the unfavorable angle of the LAD. After that, we placed an NHancer dual-lumen microcatheter on the Cx and we tried again to wire the LAD with a Sion black guide through the side hole, with no success. At this point, we had two options: to remove the jailed wire and lose its stabilizing effect to try a different guiding catheter or to try a Venture Rx torqueable microcatheter. We decided to use the second option, and we successfully wired the distal LAD with a Sion black that we exchanged with a Viperwire. We performed an OCT scan that showed the following findings: an ulcerated, calcified, and empty proximal plaque; a long segment with 180° of calcium; and a distal subocclusive lipidic plaque (Figure 5A,B). We decided to treat with orbital atherectomy with multiple applications at 80 and 120 rpm (Figure 5C). The OCT scan after the atherectomy showed deep cracking of the calcium in the proximal and mid LAD and dissection of the proximal and distal plaques (Figure 5D). After predilatation with 2.5 and 3 mm NC balloons, we stented with a 2.5 × 33 mm and 3 × 33 mm DES. The postdilatations were performed with 3 and 3.5 mm NC balloons and a KB on D1 (dissected during the LAD preparation) with a good angiographic and OCT result. The patient was discharged at home 2 days later with 60% EF and normalization of the EKG findings.

## 6. Conclusions

Coronary artery calcification is a complex anatomical and histological milieu, with different pathways that contribute to calcium deposit and calcification progression. Since no drug has shown to be effective in changing this process once it is started, proper knowledge of the underlying pathogenesis and how to diagnose and manage it is essential in contemporary coronary intervention. As part of the atherosclerotic process, extensive calcifications are becoming more common and are associated with poorer PCI outcomes if not properly addressed. To successfully achieve this goal, the use of intracoronary imaging is often mandatory in order to properly choose among the main technologies that are available today. The simple and rational algorithm for the choice of a preferential first strategy in the treatment of severely calcified coronary atherosclerosis we propose here is a good example of how we can integrate angiographic and imaging information to guide the procedure.

## Data Availability

Not applicable.
